# Bullying and Its Associated Factors among School-Aged Adolescents in Thailand

**DOI:** 10.1155/2013/254083

**Published:** 2013-02-07

**Authors:** Supa Pengpid, Karl Peltzer

**Affiliations:** ^1^ASEAN Institute for Health Development, Mahidol University, Salaya, Phutthamonthon 73170, Thailand; ^2^Department of Psychology, University of Limpopo, Turfloop Campus, Private Bag X1106, Sovenga 0727, South Africa; ^3^HIV/AIDS, STIS and TB (HAST), Human Sciences Research Council, Private Bag X41, Pretoria 0001, South Africa

## Abstract

The aim of this study were to assess bullying and its associated factors in school-going adolescents in Thailand. Using data from the Thailand Global School-Based Student Health Survey (GSHS) 2008, the prevalence of being bullied and its associated factors among adolescents (*N* = 2758) was assessed. The study found an overall prevalence of being bullied on one or more days during the past 30 days of 27.8%, 32.9% among males and 23.2% among females. The predominant forms of being bullied were among boys being hit, kicked, pushed, shoved around, or locked indoors and among girls making fun of with sexual jokes, comments, and gestures. Among boys risk factors for having been bullied were younger age (adjusted odds ratio to (AOR): 0.34; 95% confidence interval (CI): 0.18–0.65), having been in a physical fight (AOR: 3.64; 95% CI: 2.84–4.66), being physically inactive (AOR: 1.49; 95% CI: 1.04–2.15), truancy (AOR: 1.66; 95% CI: 1.13–2.45), and psychosocial distress (AOR: 2.07; 95% CI: 1.14–3.74), and among girls risk factors for having been bullied were having been in a physical fight (AOR: 2.91; 95% CI: 2.00–4.24), lack of parental bonding (AOR: 0.71; 95% CI: 0.51–0.99), and psychosocial distress (AOR: 2.37; 95% CI: 1.39–4.03). Results may inform school health programmes on the prevalence and correlates of bullying among adolescents in Thailand.

## 1. Introduction 

Bullying (occurring through interpersonal power imbalance) is a major source of victimization among adolescents which has been associated with more serious violent behaviours and may have consequences for multiple health behaviours and health outcomes [1]. The prevalence of bully victimization was examined in middle-school students in 19 low- and middle-income countries, ranging from 7.8% in Tajikistan to 60.9% in Zambia [2], and in 9 developing countries, being bullied (being bullied at least once in the preceding 30 days) was 42% among males and 39% among females [3].

Studies from low- and middle-income countries found that factors associated with being bullied include male gender [1, 2, 4], younger age [2, 5], physical fighting [1, 5, 6], mental distress [1, 2, 4, 6, 7], substance use [1–4, 7], sexual risk behaviour [1, 2], other health behaviours such as the lack of hygiene behaviour and physical inactivity [3, 5], having close friends [7], receiving parental supervision [6, 7], and being truant [4, 7]. There is a lack of studies on bully victimization among adolescents in Thailand. One study among Pattani primary schools in Southern Thailand found that 32.9% reported that they had (ever) bullied other children [8], and in a survey among lower secondary school students in Pattani province, Southern Thailand, the overall prevalence of physical bullying was found to be 18.5% [9]. Therefore, the aim of this study was to assess the prevalence of being bullied and its associated factors in school-going adolescents in Thailand. 

## 2. Methods

### 2.1. Participants and Procedures

The study involved the secondary analysis of existing data from the 2008 Thailand Global School-Based Health Survey (GSHS). Details and data of the GSHS can be accessed at http://www.who.int/chp/gshs/methodology/en/index.html. The aim of the GSHS is to collect data from students of age 13 to 15 years. The Thailand GSHS was a school-based survey of students in Grades 7, 8, 9, and 10. A two-stage cluster sample design was used to collect data to represent all students in Grades 7, 8, 9, and 10 in the country. At the first stage of sampling, schools were selected with probability proportional to their reported enrollment size. In the second stage, classes in the selected schools were randomly selected and all students the in selected classes were eligible to participate irrespective of their actual ages. The school response rate was 100%, the student response rate was 93%, and the overall response rate was 93%. Students self-completed the questionnaires to record their responses to each question on a computer scannable answer sheet. A total of 2,767 students participated in the Thailand GSHS (Ministry of Public Health). The GSHS 10-core questionnaire modules address the leading causes of morbidity and mortality among children and adults worldwide: tobacco, alcohol, and other drug use; dietary behaviors; hygiene; mental health; physical activity; sexual behaviors that contribute to HIV infection, other sexually transmitted infections, and unintended pregnancy; unintentional injuries and violence; protective factors, and respondent demographics [10, 11].

### 2.2. Measures

#### 2.2.1. Exposure to Bullying

To assess the exposure to a bullying behaviour, students were prompted with the following: “Bullying occurs when a student or group of students say or do bad and unpleasant things to another student. It is also bullying when a student is teased a lot in an unpleasant way or when a student is forced to withdraw from certain activities on purpose. It is not bullying when two students of about the same strength or power argue or fight or when teasing is done in a friendly and hilarious way.” Students were then asked the following question: “During the past 30 days, on how many days were you bullied?” Response options ranged from 1 = 0 days to 7 = all 30 days. Those reporting one or more days were considered to have been bullied. In addition, those who had been bullied were asked about how they had been bullied in the past 30 days most often.

#### 2.2.2. Health Behavior

*Physical Fights*. To assess physical fights, students were prompted with the following: “Physical fights which occurs when two or more students of about the same strength or power choose to fight each other.” Students were then asked “During the past 12 months, how many times were you in a physical fight? Response options ranged from 1 = 0 times to 8 = 12 or more times. Those reporting once or more times in a physical fight in the past 12 months were considered to be physically fighting.

*Substance Use Variables*. Tobacco use: (i) smoking cigarettes (current smoking) and (ii) The use of any other form of tobacco (current other tobacco use) in the past 30 days. Tobacco use was assessed with the question “During the past 30 days, on how many days did you smoke cigarettes (use other tobacco products?” Response options included 1 = 0 days to 7 = all 30 days. Alcohol use was assessed with the question “During the past 30 days, on how many days did you have at least one drink containing alcohol?” Response options included 1 = 0 days to 7 = all 30 days. Drug use: “During your life, how many times have you used drugs, such as methamphetamines (Yaba), ecstasy, 4 × 100, or marijuana?” (ever drugs). 

*Sexual Risk Behavior*. Sexual risk behaviour was assessed with the following question: “During your life, with how many people have you had sexual intercourse?” Two or more sexual partners in lifetime were coded as sexual risk behaviour.

*Physical Activity. *Leisure time physical activity was assessed by asking participants: The following questions “During the past 7 days, on how many days were you physically active for a total of at least 60 minutes per day?” and “During a typical or usual week, on how many days are you physically active for a total of at least 60 minutes per day?” Physical activity was defined as any activity that increases heart rate and makes one get out of breath some of the time. Physical activity can be done in sports, playing with friends, or walking to school. Some examples of physical activity are running, fast walking, biking, dancing, and football. Physical education or gym classes were not supposed to be included. According to the scoring protocol of the PACE + Adolescent Physical Activity Measure and existing guidelines, physical activity was defined as obtaining at least 60 min of physical activity per day on at least five days per week. For analysis, the number of active days “during the past week” and the number of active days “during a typical week” were averaged. 

*Hunger.* A measure of hunger was derived from a question reporting the frequency that a young person went hungry because there was not enough food at home in the past 30 days (response options were from 1 = never to 5 = always) (coded 1 = most of the time or always and 0 = never, rarely, or sometimes). 

#### 2.2.3. Psychosocial Distress Variables

*Loneliness.* “During the past 12 months, how often have you felt lonely?” (Response options were from 1 = never to 5 = always) (Coded 1 = most of the time or always and 0 = never, rarely, or sometimes).

*Anxiety or Worried*. “During the past 12 months, how often have you been so worried about something that you could not sleep at night?” (Response options were from 1 = never to 5 = always) (Coded 1 = most of the time or always and 0 = never, rarely, or sometimes).

*Sadness*. “During the past 12 months, did you ever feel so sad or hopeless almost every day for 2 weeks or more in a row that you stopped doing your usual activities?” (Response options were 1 = yes and 2 = no) (Coded 1 = 1, 2 = 0).

*No Close Friends.* “How many close friends do you have?” (Response options were from 1 = 0 to 4 = 3 or more; coded 1 = 1 and 2–4 = 0.).

#### 2.2.4. Protective Factors

Protective factors including school attendance, peer support at school, parental or guardian supervision, connectedness, and bonding.

*Truancy*. “During the past 30 days, on how many days did you miss classes or school without permission?” (Response options were from 1 = 0 times to 5 = 10 or more times) (Coded 1 = 1 or 2 to 10 or more times and 0 = 0 times).

*Peer support* at school was assessed with the question “During the past 30 days, how often were most of the students in your school kind and helpful?” *Parental or guardian supervision *was assessed with the equation “During the past 30 days, how often did your parents or guardians check to see if your homework was done?” *Parental or guardian connectedness *was assessed with the equation “During the past 30 days, how often did your parents or guardians understand your problems or worries?” And *parental or guardian bonding *was assessed with the equation “During the past 30 days, how often did your parents or guardians really know what you were doing with your free time?” Response options to these questions were from 1 = never to 5 = always, coded 1 = never or rarely and 0 = sometimes to always.

## 3. Data Analysis

Data analysis was performed using STATA software version 10.0 (Stata Corporation, College Station, TX, USA). This software has the advantage of directly including robust standard errors that account for the sampling design, that is, cluster sampling owing to the sampling of school classes. To account for the complex sampling design and to obtain accurate variance estimates, we use the “svy” estimation commands for complex survey data in STATA. Psychosocial distress was assessed across the 4 mental health measures when a student's response was indicative of distress: loneliness, anxiety or worry, sadness, and suicide plan. The number of psychosocial distress indicators was calculated by determining if students had 0, 1, 2, and 3 or 4 indicators [12]. Associations between age, health behaviour variables, psychosocial distress, protective factors, and being bullied among school children were evaluated calculating odds ratios (OR). Unconditional logistic regression was used for evaluation of the impact of explanatory variables for being bullied for boys and girls (binary dependent variables). Independent variables used in the multivariable models were age, health behaviour such as current tobacco use, psychosocial distress and protective factors such as peer support. All variables statistically significant at the *P* < .05 levels in bivariate analyses were included in the multivariable models. In the analysis, weighted percentages are reported. The reported sample size refers to the sample that was asked the target question. The two-sided 95% confidence intervals are reported. The *P* values less or equal to 5% are used to indicate the statistical significance. Both the reported 95% confidence intervals and the *P* value are adjusted for the multistage stratified cluster sample design of the study. 

## 4. Results

### 4.1. Sample Characteristics

Table 1 gives the sample characteristics of 2758 participants, mainly between 12–15 years old and 53.2% females and 46.8% males. The study found an overall prevalence of being bullied on one or more days during the past 30 days of 27.8%, 32.9% among males and 23.2% among females. Among students who were bullied during the past 30 days, 24.8% were bullied most often by making fun of with sexual jokes, comments, and gestures and 22.5% were bullied most often by being hit, kicked, pushed, shoved around, or locked indoors. Male students (31.0%) were more likely than female students (12.2%) to be bullied most often by being hit, kicked, pushed, shoved around, or locked indoors, while female students (31.0%) were more likely than male students (20.1%) to be bullied by making fun of with sexual jokes, comments, and gestures. Overall, 33.3% were in a physical fight in the past 12 months, 45.6% among males and 21.7% among females. Current smoking and current other tobacco use were reported by 8.2% and 7.2%, respectively. Current alcohol use was 14.8%, and lifetime illicit drug use 6.0%. Sexual intercourse in the past 12 months was reported by 11% of the adolescents. Substance use and sexual behaviour variables were all higher among males than females. More than a quarter (26.6%) of the participants had at least one psychosocial distress indicator, 3.4% indicated that mostly or always they felt hungry, and more than three quarters of students indicated physical inactivity (see Table 1).

### 4.2. Association with Bullying

In bivariate analyses among boys, younger age, health behaviour variables (physical fight, substance use, multiple sexual partners, physically inactive, or gone hungry), psychosocial distress, no close friends, and protective factors (truancy and lack of peer and parental support) were associated with being bullied, while, in multivariable unconditional regression analysis, younger age, having been in a physical fight, being physically inactive, truancy, and psychosocial distress were retained. In bivariate analyses among girls having been in a physical fight, truancy, lack of peer support, lack of parental bonding, and psychosocial distress were found to be associated with being bullied, while, in multivariable unconditional regression analysis, having been in a physical fight, lack of parental bonding, and psychosocial distress were retained in the model (see Table 2).

## 5. Discussion

The study found an overall prevalence of being bullied on one or more days during the past 30 days of 27.8%, 32.9% among males and 23.2% among females in school-going adolescents in Thailand. A study of bullying in eight African countries found in most countries higher rates of bullying than found in this study [1], ranging from 25% in Tanzania to 63% in Zambia, and among 9 developing countries being bullied (at least once in the preceding 30 days) was 42% among males and 39% among females [3].

This study found that males were more likely to have been victims of bullying compared to females, which conforms to some other studies [1, 4, 5, 7]. Further, the study found that predominant forms of bullying among boys were being hit, kicked, pushed, shoved around, or locked indoors and among girls making fun of with sexual jokes, comments, and gestures. Similar gender distributions regarding verbal and physical forms of bullying have been reported by Volk et al. [13]. Unlike this study, in a study among school-going adolescents in Chile, the predominant type of bullying was made fun of because of how the body or face looks [5].

The study found in the multivariable analysis among boys that younger age, having been in a physical fight, being physically inactive, truancy, and psychosocial distress were associated with being bullied and among girls having been in a physical fight, lack of parental bonding, and psychosocial distress were associated with being bullied. Some of these factors have been reported to be associated with bully victimization, that is, younger age [5], physical fighting [1, 5, 6], and mental distress [1, 2, 4, 6, 7]. The association between physical inactivity and bully victimization among boys can be perceived that physically active students are fit and may therefore be able to protect themselves [3]. Physical activity or exercise may have psychological benefits by improving social skills [14] and physical fitness and could contribute to the prevention of bully victimization among boys. Male school children who may be bullied may miss classes in order to avoid being victimized. The finding in the current study that missing classes was associated with victimization from bullying has also been reported in China and Zambia [4, 7].

This study did not find any association between bully victimization and substance use (tobacco, alcohol, and drug use), which is in contrast with a number of other studies [1–4, 7]. Further, this study did not also find an association between sexual risk behaviour [1, 2], having close friends [7], and receiving parental supervision [6, 7]. However, parental bonding was among girls protective from bully victimization. The study identified several factors including physical fighting, psychosocial distress, and lack of protective factors that increase bully victimization. Increased awareness of the high frequency of exposure to bullying and its potential health consequences such as physical fighting and psychosocial distress may lead to improvements in health promotion programmes [1]. Lack of protective factors such as truancy and parental or guardian bonding can be addressed with Check and Connect, a school-based intervention program designed to engage students in school and support regular attendance [15, 16]. Within the *check* component, risk factors are monitored, including signs of school withdrawal. The *connect* component of the model utilizes a one-on-one mentor/monitor system to establish a long-term relationship with the student, family, and school staff [16].

## 6. Study Limitations

This study had several limitations. Firstly, the GSHS only enrolls adolescents who are in school. School-going adolescents may not be representative of all adolescents in a country as the occurrence of bullying behaviour may differ between the two groups. Also we did not assess regional and urban-rural differences in bullying. As the questionnaire was self-completed, it is possible that some study participants may have missreported either intentionally or inadvertently on any of the questions asked. Intentionally missreporting was probably minimized by the fact that study participants completed the questionnaires anonymously. Furthermore, this study was based on data collected in a cross-sectional survey. We cannot, therefore, ascribe causality to any of the associated factors in the study. Future studies with a longitudinal study design are required in order to assess potential causal pathways between bully victimization and health behavior and psychosocial distress and protective factors. 

## 7. Conclusion

Bully victimization is common among school-going adolescents and is linked with physical fighting and psychosocial distress in Thailand. Results may inform school health programmes on the prevalence and correlates of bullying among adolescents in Thailand.

## Figures and Tables

**Table 1:** Sample characteristics among adolescents in Thailand, 2008.

	Total *N* = 2758 (100%)	Males *N* = 1364 (46.8%)	Females *N* = 1394 (53.2%)
Age (years)			
≤12	466 (17.0)	201 (15.6)	265 (18.2)
13	840 (29.5)	407 (30.9)	433 (28.1)
14	870 (28.7)	443 (30.3)	427 (27.2)
≥15	582 (24.9)	313 (23.2)	269 (26.5)
Bullying			
Being bullied in the past month	679 (27.8)	383 (32.9)	296 (23.2)
Among those who were bullied, the most common type of bullying			
(i) Made fun of with sexual jokes, comments, and gestures	165 (24.8)	79 (20.1)	86 (31.0)
(ii) Hit, kicked, pushed, shoved around, or locked indoors	156 (22.7)	120 (31.0)	36 (12.2)
(iii) Made fun of because of how body or face looks	78 (11.3)	25 (6.4)	53 (17.8)
(iv) Made fun of because of race and colour	51 (7.7)	28 (7.7)	23 (7.8)
(v) Made fun of because of religion	20 (2.9)	17 (4.4)	2 (0.6)
(vi) Left out of activities on purpose or ignored	17 (2.4)	9 (2.3)	8 (2.5)
(v) Bullied in some other way	194 (28.3)	107 (28.2)	86 (28.2)
Health behaviour			
Was in a physical fight in the past 12 months	931 (33.3)	617 (45.6)	314 (21.7)
Current smoking	220 (8.2)	190 (15.0)	30 (2.2)
Current other tobacco use	201 (7.2)	183 (13.4)	18 (1.3)
Current alcohol use	368 (14.8)	247 (21.2)	121 (9.3)
Lifetime illicit drug use	167 (6.0)	147 (11.1)	20 (1.3)
Two or more sexual partners in lifetime	141 (5.5)	121 (9.8)	20 (1.6)
Physical activity less than 60 min per day on at least five days per week	2073 (76.3)	914 (67.5)	1159 (84.6)
Most of the time or always hunger	94 (3.4)	63 (4.7)	31 (2.1)
No psychosocial distress indicators			
0	1939 (73.4)	935 (73.3)	1004 (73.4)
1	445 (16.9)	206 (16.6)	239 (17.3)
2	192 (7.1)	101 (8.0)	91 (6.4)
3 or 4	65 (2.5)	27 (2.0)	38 (3.0)
No close friends	94 (3.4)	62 (4.5)	32 (2.4)
Protective factors			
Truancy	467 (17.1)	317 (24.0)	150 (10.6)
Peer support	1123 (41.7)	462 (34.4)	661 (48.5)
Parental or guardian supervision	994 (35.9)	469 (35.2)	525 (36.6)
Parental or guardian connectedness	927 (34.2)	384 (28.7)	543 (39.3)
Parental or guardian bonding	1214 (45.4)	514 (38.6)	700 (51.8)

**Table 2:** Bivariate and multivariable logistic regression analysis of factors that are associated with bullying among adolescents in Thailand, 2008.

	Male		Female	
	OR^1^ (95% CI)	AOR^2^ (95% CI)	OR^1^ (95% CI)	AOR^2^ (95% CI)
Age				
≤12 years	1.00	1.00	1.00	—
13	0.85 (0.51–1.41)	0.68 (0.32–1.48)	1.16 (0.75–1.80)	
14	0.68 (0.47–0.99)*	0.63 (0.39–1.02)	1.18 (0.70–1.97)	
≥15 years	0.49 (0.31–0.78)**	0.34 (0.18–0.65)**	0.79 (0.44–1.42)	
Health behaviour				
Was in a physical fight in the past 12 months	3.71 (2.77–4.97)***	3.64 (2.84–4.66)***	3.66 (2.57–5.23)***	2.91 (2.00–4.24)***
Current smoking	1.61 (1.19–2.18)**	0.96 (0.47–1.95)	1.15 (0.42–3.16)	—
Current other tobacco use	2.66 (2.11–3.36)***	1.29 (0.62–2.68)	1.33 (0.50–3.49)	—
Current alcohol use	1.76 (1.43–2.15)***	0.83 (0.56–1.22)	1.57 (0.88–2.81)	—
Ever illicit drug use	3.31 (2.45–4.48)***	1.08 (0.39–2.94)	1.74 (0.65–4.63)	—
Two or more sexual partners in lifetime	2.59 (1.67–4.02)***	1.12 (0.39–3.63)	2.34 (0.87–6.26)	—
Physical activity less than 60 min per day on at least five days per week	1.52 (1.16–1.98)**	1.49 (1.04–2.15)*	1.02 (0.66–1.57)	—
Most of the time or always hunger	3.07 (1.72–5.49)***	2.20 (0.91–5.32)	0.90 (0.34–2.40)	—
Psychosocial distress				
None	1.00	1.00	1.00	1.00
One	1.45 (1.22–1.95)***	1.24 (0.85–1.81)	1.92 (1.29–2.87)**	1.47 (1.03–2.08)*
Two	3.02 (2.11–4.31)***	2.07 (1.14–3.74)*	2.86 (1.69–4.84)***	2.37 (1.33–4.03)**
Three or more	7.21 (2.03–25.64)**	4.13 (0.92–18.50)	7.59 (2.39–24.07)**	5.28 (1.46–19.18)*
No close friends	2.13 (1.10–4.15)*	0.55 (0.20–1.48)	1.19 (0.40–3.59)	—
Protective factors				
Truancy	2.12 (1.60–2.79)***	1.66 (1.13–2.45)*	1.65 (1.07–2.55)*	1.22 (0.75–1.99)
Peer support	0.72 (0.55–0.95)*	1.25 (0.81–1.93)	0.66 (0.50–0.86)**	0.77 (0.57–1.04)
Parental or guardian supervision	0.90 (0.64–1.26)	—	0.85 (0.60–1.22)	—
Parental or guardian connectedness	0.68 (0.51–0.91)*	0.95 (0.62–1.48)	0.86 (0.60–1.25)	—
Parental or guardian bonding	0.64 (0.51–0.81)***	0.83 (0.61–1.14)	0.60 (0.45–0.80)**	0.71 (0.51–0.99)*

^1^OR: odds ratio; ^2^AOR: adjusted odds ratio. ***P < .001; **P < .01; *P < .05.
